# Extracellular activation of Wnt signaling through epigenetic dysregulation of Wnt inhibitory factor-1 (Wif-1) is associated with pathogenesis of adrenocortical tumor

**DOI:** 10.18632/oncotarget.1889

**Published:** 2014-04-08

**Authors:** Yozo Mitsui, Hiroaki Yasumoto, Taichi Nagami, Miho Hiraki, Naoko Arichi, Noriyoshi Ishikawa, Asuka Araki, Riruke Maruyama, Yuichiro Tanaka, Rajvir Dahiya, Hiroaki Shiina

**Affiliations:** ^1^ Departments of Urology, Shimane University Faculty of Medicine, 89-1 Enya-cho, 693-8501 Izumo, Japan; ^2^ Pathology (Organ Pathology Unit), Shimane University Faculty of Medicine, 89-1 Enya-cho, 693-8501 Izumo, Japan; ^3^ Department of Surgery/Urology, Veterans Affairs Medical Center and Department of Urology, University of California, San Francisco, CA

**Keywords:** adrenocortical tumor, Wif-1, epigenetics, Wnt signaling, cyclin D1

## Abstract

Wnt/β-catenin signaling is considered to be an essential regulator of adrenocortical oncogenesis. Wnt inhibitory factor-1 (Wif-1), an extracellular regulator of Wnt signaling, is frequently down-regulated by hypermethylation of the promoter CpG. We investigated epigenetic regulation of Wif-1 and its association with adrenocortical (AC) tumor pathogenesis in light of Wnt activation. The AC tumors showed a high prevalence of Wif-1 promoter methylation and low prevalence of Wif-1 mRNA transcription as compared to the normal adrenal (NA) samples. Furthermore, a significant correlation was found between Wif-1 promoter methylation and mRNA transcription in the tumors. Either intracellular β-catenin accumulation or β-catenin mRNA transcription was significantly elevated in the AC tumors, which also showed an inverse correlation with Wif-1 mRNA transcription. Cyclin D1, a target gene of Wnt signaling, was also up-regulated in the AC tumors as compared with the NA samples. In addition, down-regulation of Wif-1was correlated with increased cyclin D1 at both mRNA and protein levels. However, despite the proposed activation of Wnt signaling in AC tumors, only 2 of 20 with intracellular β-catenin accumulation showed β-catenin mutations. Thus, genetic alterations of β-catenin and epigenetics-related Wif-1 promoter hypermethylation may be important mechanisms underlying AC tumor formation though aberrant canonical Wnt/β-catenin signaling activation.

## INTRODUCTION

Adrenocortical (AC) tumors can be classified as either a benign AC adenoma or AC carcinoma. The former is a common disorder of the adrenal glands with a prevalence of 3-10% of the general population, whereas AC carcinomas are extremely rare, with an estimated annual incidence of 1-2 cases per 1 million individuals in the United States [1, 2]. Over the past decade, research focusing on AC tumors has identified gene mutations and signaling pathways involved with pathogenesis [3]. Notably, wingless-type (Wnt)/β-catenin signaling functions as a major contributor to AC tumor formation [4].

The canonical Wnt/β-catenin pathway has been shown to play an important role in organ development, while dysregulation of Wnt signaling has been implicated as causative factor in some genitourinary malignancy cases [5-8]. The cell attachment molecules of β-catenin comprise a key component of the Wnt/β-catenin signaling pathway. Under inactivation of Wnt signaling, β-catenin is normally phosphorylated at the NH_2_-terminal residues, in which a glycogen synthase kinase 3β (GSK-3β) consensus motif is present, with the aid of a scaffolding complex composed of axin and adenomatous polyposis proteins (APCs). Subsequently, phosphorylated β-catenin is subject to degradation by the ubiquitin-proteasome system. Under activation of Wnt signaling with the cooperation of Wnt proteins bound to their frizzled transmembrane receptors, the functional loss of GSK-3β is a common event, namely, failure to phosphorylate β-catenin. Thus, non-phosphorylated forms of β-catenin can accumulate in the cytoplasm, enter the nucleus, and finally activate the Wnt target genes *c-myc* and *cyclin D1* as a transcriptional activator with the aid of TCF/LEF family proteins [9-11].

Active involvement of β-catenin has been postulated in the pathogenesis of AC tumors by a number of gene profiling studies [12-16]. They noted that abnormal cytoplasmic and nuclear accumulation of β-catenin were found in approximately 24-40% of AC adenomas and 30-80% of AC carcinomas, probably attributable to activated mutation of β-catenin. However, despite the lack of definite β-catenin mutations, the majority of AC tumors (70-85%) in those studies were found to be positive for β-catenin accumulation, which suggests an additional mechanism of Wnt activation involving stabilization of β-catenin.

The secreted frizzled-related protein (sFRP), known to be a Wnt antagonist, inhibits the Wnt signaling pathway by direct binding to Wnt molecules instead of frizzled molecules [17]. In a recent study of a cohort of human cancers targeting a variety of tissue types, down-regulation of sFRP was found in AC tumors [18]. Thus, functional loss of Wnt antagonists such as the sFRP class may contribute to activation of the Wnt pathway in these tumors.

Wnt inhibitory factor-1 (Wif-1), a sFRP family protein initially identified in the human retina [19], has been shown to be down-regulated in bladder and kidney cancer, and its expression is inversely correlated with promoter CpG hypermethylation [8, 20]. In bladder cancer, down-regulation of Wif-1 was found to be associated with enhanced expression of Wnt target genes such as c-myc and cyclin D1 [8]. The aim of the present study was to assess whether activation of the Wnt signaling pathway caused by epigenetics-related Wif-1 dysregulation has an association with the pathogenesis of AC tumors.

## RESULTS

### Correlation of Wif-1 with Wnt target genes

**Figure 1:**
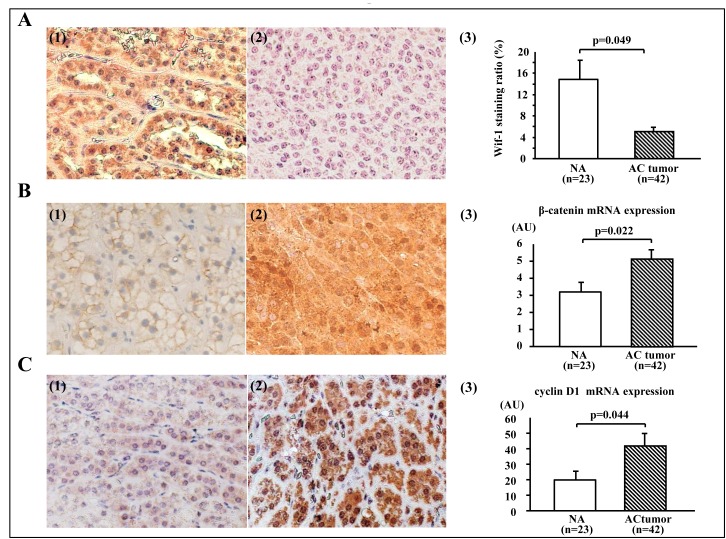
Expressions of Wif-1, β-catenin, and cyclin D1 in adrenocortical tumors A (1). Representative immunostaining of Wif-1 in normal adrenal gland (NA) sample (×200). Strong cytoplasmic and/or membranous staining of Wif-1 was observed in the NA samples. A(2). Representative immunostaining of Wif-1 in adrenocortical (AC) tumor (×200). No membranous or cytoplastic staining of Wif-1 was observed in the AC tumors. A(3). Strong cytoplasmic and/or membrane staining of Wif-1 was more common in the NA samples than in the AC tumors (p=0.049). B(1). Representative immunostaining of β-catenin in the NA sample (×200). Membranous staining of β-catenin was observed in the NA samples. B(2). Representative immunostaining of β-catenin in AC tumor with negative Wif-1 expression (×200). Strong nuclear and cytoplasmic staining of β-catenin was observed in the AC tumors. B(3). AC tumors showed a higher level of β-catenin expression at the mRNA transcription level in comparison with NA samples (p=0.022). C(1). Representative immunostaining of cyclin D1 in NA sample (×200). Weak cytoplasmic staining of cyclin D1 was observed in the NA samples. C(2). Representative immunostaining of cyclin D1 in AC tumors with negative Wif-1 expression (×200). Strong nuclear and moderate cytoplasmic staining of cyclin D1 was observed in the AC tumors. C(3). Cyclin D1 mRNA transcription was higher in the AC tumors than in the NA samples (p=0.044)

**Figure 2:**
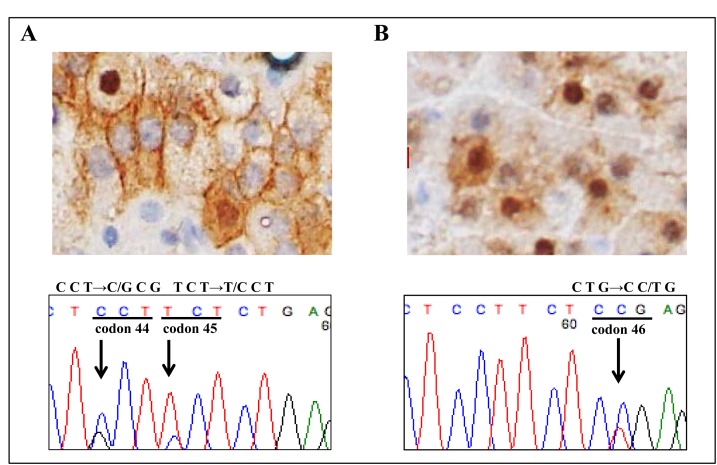
Mutation analysis of *β-catenin* gene A molecular alteration in exon 3 of the *β-cateni*n gene was observed in 2 non-secreting adrenocortical adenomas. A. β-catenin immunostaining showed strong nuclear accumulation (×400). Nucleotide sequencing of exon 3 of β-catenin showed a T→G transition (L46P) in case 1. B. A small subset of tumor cells showed strong nuclear accumulation of β-catenin (×400). Nucleotide sequencing of exon 3 of β-catenin showed a C→G transition (P44A) and T→C transition (S45P) in case 2.

**Figure 3:**
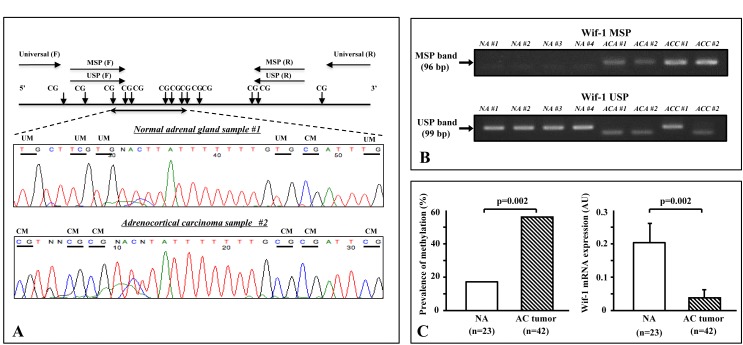
Methylation analysis of clinical samples A. Schema of Wif-1 promoter, locations of the primers, and typical bisulfite DNA sequencing in normal adrenal gland (NA) samples and adrenocortical (AC) tumors. The universal primers (F, forward; R, reverse) used did not contain any CpG sites within the primer sequence. The F and R methylation-specific PCR (MSP) primers contained 3 and 1 CpG sites, respectively, with unmethylation-specific PCR primers designed in the same manner. In the NA samples, the majority of cytosines within the CpG sites were completely converted to thymines (unmethylated) after bisulfite modification. On the other hand, the majority of cytosines remained virtually unchanged after bisulfite modification in the AC tumors. Horizontal bar, CpG sites; UM, unmethylation; CM, complete methylation. B. Representative results of methylation-specific PCR (MSP) and unmethylation-specific PCR (USP) of Wif-1 promoter in clinical samples. Top and bottom show MSP and USP bands, respectively, from the same samples. AC adenomas samples (ACA #1 and #2) and AC carcinoma samples (ACC #1 and #2) showed an MSP band, whereas NA samples (NA #1 to #4) showed a USP band and no MSP band. C. Promoter methylation of Wif-1 was found in 4 of the NA samples and 24 of 42 of the AC tumors. There was a significant difference regarding the incidence of Wif-1 methylation between NA and ACT (p=0.002). In addition, the level of Wif-1 mRNA transcription was significantly lower in the AC tumors than in the NA samples (p=0.002).

A representative Wif-1 immunostaining pattern is shown in Figure 1A. Strong cytoplasmic and/or membrane staining of Wif-1 was more common in the normal adrenal gland (NA) samples than in the AC tumor specimens (Fig. 1A-3, p<0.05). Also, a positive correlation was found between mRNA transcription and protein levels of Wif-1 (p<0.0001, data not shown). As for β-catenin immunostaining (Figs. 1B-1 and -2), intracellular β-catenin accumulation was positive in 47.6% (20/42) of the AC tumors, whereas only 4.3% (1/23) of the NA samples were positive. Of the 20 β-catenin positive AC tumors, 6 were also positive for nuclear β-catenin accumulation (Table 1). At the mRNA transcript level, the AC tumors showed a higher level of β-catenin expression in comparison with the NA samples (Fig. 1B-3). Furthermore, overexpression of cyclin D1 was positive in 31.0% (13/42) and 17.5 % (4/23) of the AC tumors and NA samples, respectively (Fig. 1C). Likewise, cyclin D1 mRNA transcription was enhanced in AC tumors as compared to the NA samples (Fig. 1C-3, p<0.05).

**Table 1:** Clinical, pathological, molecular features in AC tumors

Patient ID	Histology	Sex	Age (years)	Functional status	Tumor volume (g)	Tumor stage	IHC status of β-catenin (nuclear/cytoplasm)	β-catenin mutation	Wif-1 methylation
1	ACC	M	70	NF	357	IV	Neg.	No	Yes
2	ACC	F	33	NF	110	III	Pos. (nuclear)	No	Yes
3	ACC	M	68	NF	4	I	Pos. (cytoplasm)	No	Yes
4	ACA	F	58	NF	2	**-**	Pos. (cytoplasm)	No	No
5	ACA	F	63	NF	7	**-**	Pos. (cytoplasm)	No	No
6	ACA	F	47	NF	6	**-**	Pos. (cytoplasm)	No	No
7	ACA	M	73	NF	25	**-**	Neg.	No	Yes
8	ACA	F	50	NF	8	**-**	Neg.	No	Yes
9	ACA	F	56	NF	10	**-**	Pos. (cytoplasm)	No	Yes
10	ACA	M	48	NF	50	**-**	Pos. (nuclear)	L46P	Yes
11	ACA	M	71	NF	17	**-**	Neg.	No	Yes
12	ACA	M	70	NF	12	**-**	Pos. (cytoplasm)	No	No
13	ACA	M	67	NF	9	**-**	Pos. (nuclear)	P44A S45P	No
14	ACA	M	60	NF	1	**-**	Pos. (cytoplasm)	No	Yes
15	ACA	M	62	NF	30	**-**	Pos. (cytoplasm)	No	Yes
16	ACA	M	50	NF	4	**-**	Pos. (cytoplasm)	No	No
17	ACA	F	46	NF	6	**-**	Pos. (cytoplasm)	No	Yes
18	ACA	F	70	NF	23	**-**	Neg.	No	Yes
19	ACA	F	80	NF	20	**-**	Pos. (nuclear)	No	Yes
20	ACA	F	45	ALD	10	**-**	Neg.	No	Yes
21	ACA	M	52	ALD	28	**-**	Neg.	No	No
22	ACA	F	57	ALD	7	**-**	Pos. (cytoplasm)	No	No
23	ACA	F	55	ALD	13	**-**	Pos. (cytoplasm)	No	No
24	ACA	M	53	ALD	9	**-**	Neg.	No	No
25	ACA	F	64	ALD	14	**-**	Pos. (nuclear)	No	Yes
26	ACA	F	63	ALD	9	**-**	Neg.	No	Yes
27	ACA	F	69	ALD	6	**-**	Neg.	No	No
28	ACA	M	32	ALD	23	**-**	Neg.	No	No
29	ACA	F	42	ALD	6	**-**	Neg.	No	Yes
31	ACA	M	52	ALD	7	**-**	Pos. (cytoplasm)	No	Yes
31	ACA	F	58	ALD	5	**-**	Neg.	No	No
32	ACA	F	65	ALD	5	**-**	Neg.	No	No
33	ACA	M	62	ALD	19	**-**	Neg.	No	No
34	ACA	F	39	ALD	5	**-**	Neg.	No	Yes
35	ACA	F	46	ALD	11	**-**	Neg.	No	No
36	ACA	M	59	COR	27	**-**	Neg.	No	No
37	ACA	F	56	COR	42	**-**	Neg.	No	Yes
38	ACA	F	51	COR	7	**-**	Neg.	No	Yes
39	ACA	F	62	COR	8	**-**	Pos. (cytoplasm)	No	Yes
40	ACA	F	21	COR	15	**-**	Neg.	No	No
41	ACA	M	76	COR	6	**-**	Neg.	No	Yes
42	ACA	F	58	COR	5	**-**	Pos. (nuclear)	No	Yes

ACC, Adrenocortical carcinoma; ACA, adrenocortical adenoma; M, male; F, female; NF, nonfunctioning; ALD, aldosterone; COR, cortisol; IHC, immunohistochemistry.

### Mutational analysis of β-catenin gene

A genetic alteration encompassing exon 3 of the *β-cateni*n gene was found in only 2 of the 20 AC tumors that showed intracellular β-catenin accumulation (Table 1). These 2 tumors harboring a *β-cateni*n mutation were both the non-secreting phenotype of AC adenoma. As shown in Figure 2A, there were 2 different mutations, resulting in proline to alanine (P44A) and serine to proline (S45P) in 1 of the tumors, while Figure 2B shows a mutation resulting in leucine to proline (L46P) that was found in the other.

### Analysis of methylation in clinical samples

The primer location and its sequences for PAN, methylation-specific (MSP), and unmethylation-specific PCR (USP), as well as typical bisulfite DNA sequencing of the NA samples and AC tumors in relation to primer location are shown in Figure 3A. In the NA samples, the majority of cytosines within the CpG sites were completely converted to thymines (unmethylated) after bisulfite modification, indicating that methylation of Wif-1 by promoter CpG was predominantly negative (Fig. 3A; middle panel). On the other hand, the majority of cytosines remained virtually unchanged after bisulfite modification in the AC tumor samples (Fig. 3A; lower panel).

Representative MSP and USP bands are shown in Figure 3B. Twenty-four of 42 (57.1%) of the AC tumors were found to be positive for Wif-1 methylation, while only 4 of the 23 (17.4%) NA samples were positive (Fig. 3C; left panel). It is interesting to note that sample numbers NA#1 and ACC#2 were identical to the bisulfite DNA sequencing shown in Figure 3A, suggesting that our findings of methylation analysis using the combination of MSP and USP were consistent with the results of bisulfite DNA sequencing. As shown in Figure 3C (right panel), the expression of Wif-1 mRNA transcription was significantly lower in the AC tumors than in the NA samples (p = 0.002). In addition, a significantly inverse correlation was found between Wif-1 mRNA transcripts and methylation of the Wif-1 promoter in the AC tumors (p=0.003, data not shown). No significant association of Wif-1 methylation was found with tumor histology or tumor volume.

### Relationship of β-catenin with Wif-1 and Wnt target genes

**Figure 4:**
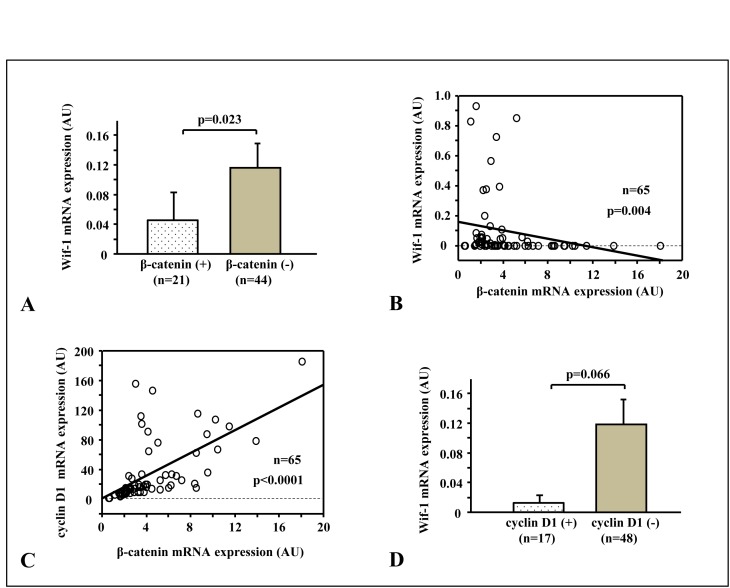
Relationships of β-catenin with Wif-1 and Wnt target genes A. Specimens with intracellular β-catenin accumulation (β-catenin+) showed lower Wif-1 mRNA transcription than those with negative accumulation (p=0.023). B.There was a significantly inverse correlation between Wif-1 mRNA expression and β-catenin mRNA expression (p=0.004). C. β-catenin mRNA expression was positively correlated with cyclin D1 mRNA expression (p<0.0001). D. Wif-1 mRNA transcription was decreased in the samples with cyclin D1 over-expression as compared to those without that over-expression (p=0.066).

The central constituent of Wnt signaling is β-catenin, thus the relationships of β-catenin with other Wnt-related genes were evaluated. In the total series, samples with intracellular β-catenin accumulation showed lower levels of Wif-1 mRNA transcription as compared to those with negative accumulation (p<0.05) (Fig. 4A). Likewise, constitutive mRNA transcription of β-catenin, as shown by RT-PCR, was inversely correlated with the mRNA transcription level of Wif-1 (Fig. 4B), which was also well correlated with cyclin D1, a downstream regulator of Wnt signaling (Fig. 4C). As shown in Figure 4D, Wif-1 mRNA transcription was decreased in the samples with cyclin D1 over-expression as compared to those without that over-expression.

## DISCUSSION

Wnts are a family of secreted glycoproteins that regulate a wide variety of biological processes, including embryonic development, cell proliferation, cell migration, tumor suppression, and oncogenesis. Previous studies have shown that activation of Wnt signaling can promote the pathogenesis of various human cancers, including AC tumor development through a canonical pathway, which is also called the Wnt/β-catenin signaling pathway [12-16, 21]. Activated Wnt signaling accelerates cell proliferation and further oncogenesis, thus recruiting β-catenin into a transcriptional activation complex with the aid of TCF/LEF.

Among AC tumors, it has been reported that approximately 24-40% of AC adenomas and 30-80% of AC carcinomas have abnormal intracellular accumulation of β-catenin [12-16]. In the present study, we found abnormal β-catenin staining in approximately half of the AC tumors (adenoma 46.1%, carcinoma 66.7%). In addition, levels of immunoreactive protein as well as mRNA transcription of β-catenin were found to be higher in the AC tumors than the NA samples. Furthermore, mRNA transcription of both cyclin D1 and β-catenin was at a significantly higher level in the AC tumors as compared to the NA samples, indicating that cyclin D1 is a downstream effector of Wnt signaling closely related to intracellular accumulation of β-catenin. These findings are in line with the notion of possible involvement of activated Wnt signaling in adrenal oncogenesis.

Activation of a β-catenin exon 3 mutation is one of the most frequent genetic alterations seen in association with activated Wnt signaling. However, the majority of AC tumors are known to accumulate intracellular β-catenin despite the lack of genetic mutations of β-catenin [12-16]. Indeed, in our series, only 2 of 20 (10%) AC tumors with abnormal β-catenin staining possessed β-catenin mutations. In addition, bi-allelic inactivation of APC is rarely found in sporadic AC tumors, though patients with familial adenomatous polyposis APC mutation are common [22]. Therefore, molecular events beyond the genetic mutations involving the β-catenin or APC gene are thought to affect Wnt signaling activation in a significant proportion of AC tumors. On the other hand, extracellular activation of Wnt signaling has been shown to be regulated at multiple levels by proteins such as sFRP, while decreased expression of sFRP related to β-catenin activation has been reported in various human carcinomas including AC tumors [23, 24]. In order to resolve the discrepancy between the low prevalence of genetic mutations affecting β-catenin or APC and highly activated Wnt signaling in AC tumors, we speculate that canonical Wnt signaling involving adrenal oncogenesis is controlled by an upstream regulator of the Wnt pathway.

Dysregulation of Wnt antagonists such as Wif-1 comprises an alternative mechanism underlying aberrant activation of the Wnt signaling pathway. In bladder and kidney cancer, epigenetic promoter CpG hypermethylation of Wif-1 is attributable to its down-regulation [8, 20]. In the present study, CpG methylation of Wif-1 was more common in the AC tumors than NA tissues (Fig. 1B), while Wif-1 mRNA transcription was conversely up-regulated in the latter (Fig. 1C). Based on these findings, we considered that promoter CpG methylation is a potential regulator of *Wif-1* gene expression in AC tumors. Furthermore, we found an inverse correlation between mRNA transcription levels of β-catenin and Wif-1 in AC tumors, which indicates that intracellular β-catenin accumulation accelerating Wnt signaling is related to epigenetics-associated down-regulation of Wif-1, but not to posttranslational modification of β-catenin. Therefore, it is quite possible that epigenetics-related functional impairment of Wif-1 may lead to constitutive β-catenin activation and subsequent adrenal oncogenesis via cyclin D1 activation.

As for the other Wnt target genes of c-myc, there were no significant differences in regard to c-myc mRNA transcription between the AC tumors and NA samples (data not shown). Previous studies have suggested that these tumors might represent a unique class characterized by low c-myc expression [23, 25], which appears to be compatible with our results.

Recent studies of gene regulation at the posttranslational level have investigated microRNAs, which contribute to oncogenesis in various carcinomas via activation of the Wnt signaling pathway [26-29]. In pigmented nodular-adrenocortical disease, the association of miR-449 with dysregulation of WNT1-inducible signaling pathway protein 2 has been discussed [29], while the probability of miR-29s in non-small-cell lung cancer as a mechanism of escape from epigenetic regulation of Wif-1 has also been noted [30]. In the near future, it is expected that a novel molecular approach will shed light on these issues by addressing the dysregulation of Wnt/β-catenin activity in AC tumors.

A major limitation of the present study is the relatively small number of specimens examined, especially AC carcinomas. Dysregulation of the canonical Wnt/β-catenin signaling pathway may be a minimum requirement in the initial step of adrenal tumorigenesis, though that appears to be insufficient for development of malignant transformation [4]. Other genetic aberrations such as insulin-like growth factor 2 dysregulation may be required for driving AC tumor cells towards a more malignant phenotype [16, 31, 32].

In conclusion, this is the first clinical study of inactivation of the *Wif-1* gene via epigenetic pathways in AC tumors. In addition to genetic alterations of β-catenin, the epigenetics of promoter CpG hypermethylation of Wif-1 may be an important mechanism associated with the initial process of adrenal oncogenesis via aberrant canonical Wnt/β-catenin signaling activation. Although further research is required to verify our findings, our report is expected to contribute to a better understanding of the pathogenesis of AC tumors.

## METHODS

### Samples

Tissue samples were obtained from 42 patients with various types of AC lesions, including 39 AC adenomas (16 non-secreting, 16 aldosterone-secreting, 7 cortisol-secreting) and 3 AC carcinomas. Clinical and pathological features are described in Table 1. Also, 23 NA tissues which did not contain adrenal medulla tissues were obtained from patients undergoing a radical nephrectomy or nephroureterectomy, and used as the control group. The NA tissues did not contain adrenal medulla tissue. Current guidelines for diagnosis of AC adenoma and AC carcinoma were applied for histological evaluations of the AC samples, [33] and all of the adenomas had a Weiss score <2. The mean age of the patients who supplied tumor samples was 56 years (range, 21-80 years) and the female/male ratio was 25/17. Tissue samples were fixed in 10% buffered formalin (pH 7.0) and embedded in paraffin wax, then 5-μm sections were obtained and subjected to H&E staining for histologic evaluation. Written informed consent was obtained from each patient for molecular analysis of the resected specimen, and study was approved by the local ethnical committee of the Shimane University Faculty of Medicine in accordance with the 1975 Declaration of Helsinki.

### Nucleic acid extraction

Genomic DNA and total RNA were isolated from all formalin-fixed paraffin-embedded AC sample tissues using a QIAamp Tissue kit (Qiagen, Valencia, CA) and RecoverAll™ Total Nucleic Acid Isolation kit (Ambion, Inc., Austin, Texas, USA), respectively. RNA pellets obtained after isopropanol and ethanol precipitation were dried, re-suspended in 25 μL of RNase-free water, and stored in aliquots at -80°C until reverse transcription was performed. The concentrations of DNA and RNA were determined with a spectrophotometer, and the integrity of those measurements was checked by gel electrophoresis.

### cDNA preparation and gene quantification

Using 1 μg of RNA, 0.5 μg of oligo-dT primer, and 0.5 units of RNase inhibitor, cDNA was constructed using reverse transcriptase (Promega). The mRNA transcript levels of Wif-1, β-catenin, c-myc, and cyclin D1 were measured by a StepOne RT-PCR system (Applied BioSystem), with GAPDH utilized as the reference. The reaction protocol recommended by the manufacturer (Applied BioSystem) was strictly followed. For each run, a standard curve was generated using a serial dilution of the external standard. The expression level was calculated as the ratio of the target gene to that of GAPDH.

### Immunohistochemical analysis

Immunostaining of Wif-1, β-catenin, and cyclin D1 was performed using 5-μm-thick consecutive sections obtained from paraffin-embedded materials. Slides were prepared with antigen retrieval using citrate buffer (10 mmol/L, pH 6.0), then incubated for 12 hours with mouse monoclonal antibody for Wif-1 (1: 50, R&D Systems), β-catenin (1: 500, BD Biosciences), and cyclin D1 (1: 50, Santa Cruz Biotechnology). For a negative control, the primary antibody was replaced with non-immune serum. DAB (Sigma-Aldrich) was used as the chromogen and counterstaining was performed with hematoxylin. For β-catenin expression, cytoplasmic/nuclear staining was considered to indicate a positive reaction and staining intensity was not scored, according to a previously reported method [12]. Nuclear Cyclin D1 expression was evaluated based on the proportion of positive nuclei to all normal or tumor cells [i.e., uniformly positive (+ indicates higher expression group/>^20^% positive nuclei) and negative (- indicates lower expression group/0-20% positive nuclei)], as previously described [8]. For Wif-1 expression, cytoplasmic and membrane expressions were analyzed according to the proportion of positive cells using ImageJ software (http://rsb.info.nih.gov/ij).

### Mutation analysis of the *β-catenin* gene

The coding region of exon 3 of β-catenin was analyzed, using a previously reported technique [5]. Briefly, primers for PCR were designed to amplify a 134-bp fragment of exon 3 of the *β-catenin* gene encompassing the region for phosphorylation sites, which is considered to be the hotspot of *β-catenin* mutation. The primer sequences and PCR conditions are shown in Table. 2. The amplified PCR products were directly sequenced according to the manufacturer's instructions (Applied Biosystems, Foster City, CA). Mutations were verified in both sense and ant-sense directions.

**Table 2:** Primer sequences and PCR conditions

	Sense primer (5'-3')	Antisense primer (5'-3')	Anneal temperature (°C), PCR cycles	Product size (bp)
MSP primers				
Univiersal				
Wif-1 PAN	GTTAGTTTTGTTAGTTTTA	CTAAATACCAAAAAACCTAC	48, 45	149
Methylated				
Wif-1 MSP	TTTTGTCGTTTTTATTTTCGTTC	AAAAAAACAAACAAAACGCG	52, 45	96
Un-methylated				
Wif-1 USP	GTTTTTGTTGTTTTTATTTTTGTTT	AAAAAAAACAAACAAAACACAA	52, 45	99
RT-PCR primer				
β-catenin (mutation analysis)	AAGCGGCTGTTAGTCACTGG	CAGGACTTGGGAGGTATCCA	52, 45	134

Note: Boldface indicates the differences between modified sequence. Underline indicates the changes between methylated and un-methylated sequences after bisulfite modification.

### Methylation analysis

Genomic DNA (100 ng) was modified with sodium bisulfite using a commercial kit (Invitrogen Life Technologies, San Diego, CA). Based on the functional promoter sequence of the Wif1 gene [34], methylation- and unmethylation-specific primers were designed using the MethPrimer program (http://itsa.ucsf.edu/~urolab/methprimer). As shown in Table 2 and Figure 3A, the first set of universal primers covers no CpG sites in either the forward or reverse primer, and amplifies the 149-bp DNA fragment of the Wif-1 promoter region containing 13 CpG sites. Then, a second round of nested MSP or USP was done using universal PCR products as templates. According to a previously reported method [8, 35], the primer sequences for MSP and USP of the Wif-1 gene promoter were designed to include 11 CpG sites in this region (Fig. 3A). The primer sequences and PCR conducts (product size, PCR cycles) are shown in Table 2. In each assay, the absence of a DNA template served as a negative control. The MSP and USP products were analyzed by 2% agarose gel electrophoresis. For bisulfite DNA sequencing, 1 μl of bisulfite-modified DNA was amplified using a pair of universal primers in a total volume of 20 μL. Direct bisulfite DNA sequencing of the PCR products was done according to the manufacturer's instructions (Applied Biosystems, Foster City, CA).

### Statistical analysis

All data were analyzed using the StatView V statistical package (SAS Institute, Inc., Cary, NC). Statistical analysis was done using the Mann-Whitney test. Non-parametric data were analyzed by a chi-square test. Correlations between 2 continuous variables were analyzed by Spearman rank correlation. A P value of less than 0.05 was considered to be statistically significant.
